# Analysis of the vulnerability estimation and neighbor value prediction in autonomous systems

**DOI:** 10.1038/s41598-022-13613-3

**Published:** 2022-06-08

**Authors:** Nematullo Rahmatov, Faisal Saeed, Anand Paul

**Affiliations:** School of Computer Science and Engineering, Kyungpook National University, Daegu, 41566 Daegu, South Korea

**Keywords:** Mathematics and computing, Computer science

## Abstract

The security within autonomous systems (AS)s is one of the important measures to keep network users safe and stable from the various type of Distributed Denial of Service (DDoS) attacks. Similar to the other existing attack types Internet control message protocol (ICMP) based attacks are remained open challenge on the Internet environment. In this study, we have proposed a method to estimate the vulnerability of 600 AS provider edge (PE) routers by sending ICMP packets and predicted AS neighbor values using least square regression (LSR) approach. The results of our study show that 265 AS PE routers are vulnerable due to ICMP flood attack from the 600 ASs which were analyzed. Additionally, we have predicted that about 60% of total AS neighbors will be reduced in the next 3 years. Our results indicate that some ASs still did not deploy the firewall system in the boundary of their networks. Similarly, we also observed that the majority of ASs which expected to have less neighbor values in the next 3 years is due to change their routing paths to find adjacent paths.

## Introduction

**Figure 1:**
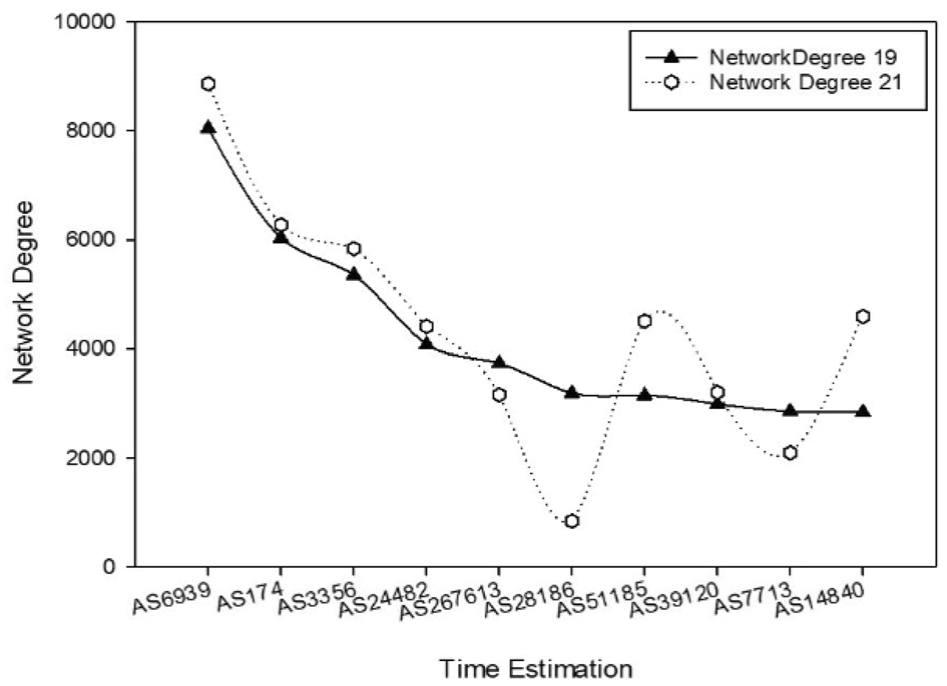
Dynamics of global autonomous system (AS) neighbors from 2019 to 2021.

One of the main terminologies for analyzing the Internet ecosystem is the autonomous system (AS). Recently, the total number of ASs worldwide is 109,437^1^. ASs play a significant role in displaying an actual distribution of Internet organizations or Internet service providers (ISPs). However, in other sentence, ASs are compounds of different types of network devices in different locations of the globe. According to the concept of a global Internet-routing policy, the routing system is divided into two types: intradomain routing policy and interdomain routing policy. An interdomain routing policy is based on border gateway protocol (BGP)^2^. BGP is being employed since 1994 to exchange routing and access data among ASs^3^. As global AS numbers are rapidly increasing, it will be necessary to analyze, monitor, and predict internet characteristics. Due to the network measurement (NM) research community, unknown Internet characteristics and rapid changes have been discovered. Compared with the recent studies in NM area in the last 3 years, the new number of ASs reached 30,000. As shown in Fig. 1, it was observed that in the last 3 years, 1522 new ASs were connected to aforementioned 10 ASs while 3686 ASs disconnected or changed their network neighbors^2^. Besides the rapid increase in AS numbers, there are many changes in the routing policy system.

This study identified these changes during the experiment based on active monitoring. One of the main goals of this study is to estimate the provider edge (PE) router IP addresses that are vulnerable in the network boundary of the different types of ASs. The different types of ASs in this study contain 150 Universities around the globe, 150 Mobile Companies, 150 Internet Exchange Point (IXP), and 150 Large ISPs. A total of 600 ASs is collected from all continents to classify AS types and discover novel changes in global AS routing policy. PE routers are connected to upstream ISP routers called customer edge (CE) routers to provide internet access to several local area networks (LANs) within AS. Another mandatory specification of PE router or AS edge router is security concern. The main importance of identifying PE router’s IP addresses of any targeted AS is estimating the network vulnerability level of that AS. Network vulnerability level is essential in any AS types to safeguard PE routers that are in the network boundary. In some ASs, firewall software is not installed in the PE router, which can be the reason for distributed denial of service (DDOS) attacks in the entire AS. Especially, there is a high risk of Mirai malware in the network devices within AS^4^. Internet ecosystems are designed in a very complex manner. It consists of hundreds of thousands of ASs^1^. Due to the rapid change in Internet AS characteristic such as routing strategy, AS numbers, increasing number of AS network degrees, and AS customer cone, it is important to discover and investigate it recurrently^2,5^. According to CIDR report, there will be an increase or decrease in the AS number yearly or even less^6–8^. In 2017, six types of AS ranking schemes were discovered by Tozal et al.^9^; however, in 2021, as AS numbers increased, not many novel studies have been conducted. This study hopefully will be a good source for network practitioners, designers, and NOC Engineers in ISP to understand the novel characteristics of Internet AS and upcoming challenges. NM is an effective area of research for analyzing AS novel characteristics and discovering previously nonexisting problems. As in many cases, network organizations install a software in the PE router called firewall. In previous study the author identified three problems to not being analogous to routing with combined active and passive monitoring while sending traceroute from the source AS to target ASs^10^. Due to frequent changes in AS routing policy, we want to examine if any other changes existed in the novel AS routing policy. The primary goal of this study is to estimate the number of ASs that are vulnerable due to internet control message protocol (ICMP) among the four types which were categorized during the experiment. By sending the ICMP probe packet to the domain name system (DNS) server of each target AS, we wanted to identify whether the PE router’s IP address is visible or blocked by a firewall program. PE routers are an essential component in any type of AS. Additionally, it must be protected using a firewall software for scanning incoming traffic from the neighboring ASs; else, there is a high possibility of the AS being attacked by network intruders from external networks. This study has diagnosed whether the DNS servers are active or not through round trip time (RTT) of target AS DNS servers. Only by knowing the active DNS servers whether it is effective to send reliable ICMP packet and identify PE routers vulnerability. RTT is a combination of two propagation time and

1 $$\mathrm{ RTT }=\mathrm{ T}{p}_{1} +\mathrm{ T}{p}_{2} ,$$

where T${p}_{1}$ is the first message time send from the sender side and T${p}_{2}$ is the second message time from the receiver side. Troubleshooting via RTT is mainly used by network operation engineers while troubleshooting their target networks. One of the challenges in this study is identifying reliable global DNS IP addresses of randomly selected AS. Usually, the DNS server is constantly in ON mode, unlike other servers in the ISP. However, requesting global DNS addresses from the Center of Applied INTERNET Data Analysis (CAIDA) also becomes a challenging task. For the experimental part, the global DNS addresses were requested several times from CAIDA; however, there were no clear answers. However, using web IP addresses instead of DNS IP addresses is not an effective strategy for discovering PE router IP addresses of the target AS, because it does not constantly keep web servers in the target ASs. Generally, for DNS IP addresses and web IP addresses of any ASs had the problems of firewall system blocking and AS path prepending while searching IP addresses^10^. However, if the firewall blocks several hops in the target AS while sending ICMP command via traceroute tool, deriving accurate RTT information and PE router IP addresses of the target AS becomes impossible. The main contributions of this paper include:

- It was analyzed ASs that are vulnerable while sending Internet Control Message Protocol (ICMP) to their PE routers among the four AS types which were categorized in this paper.
- It was predicted 600 AS neighbor values for the next three years using LSR statistical approach.

The remainder of this study is organized as follows. “Related works” section presents related works on Internet AS characteristics and future challenges. It also describes a number of literature reviews conducted in network measurement and algorithms used to solve the challenges. “Proposed methods” section presents the proposed methodologies for AS PE router vulnerability estimation and the AS neighbor values prediction. “Experiments and results” section presents the experiential results and analysis of the proposed method. Finally, “Conclusion” section presents the conclusion and provides a path for future research.

## Related works

Several research works have examined the internet characteristics of internet AS and identified threat types on internet AS^11–15^. Bakhaliev et al.^16^ investigated path dynamics for end-to-end paths and path segments using two months of measurement data from the RIPE Atlas platform. They observed that 78% of the end-to-end routes have at least two alternative AS paths starting from 3380 ASs. The result shows that individual ASs have constant paths internally and end-to-end paths are more dynamic. Similarly, Canbaz et al.^17^ investigated the network characteristics of 19,614 backbone Ass, which provide peering connectivity to another customer ASs. Datasets for router-level probes were gathered from a variety of public internet topology measurement platforms. The proposed method’s results show that a significant number of AS topologies are network-size independent and resemble disassortative network topologies. The proposed solution has the advantage of gathering data from various public platforms. Nur et al.^18^ proposed a cross AS (X-AS) Internet topology map using common network tools, such as traceroute, ping, and BGP data collector. They projected that their proposed method would be feasible into the Internet’s structural and operational characteristics, thereby improving current and future IP stack protocols. They also asserted that their method has the potential to improve networking infrastructure, synthetic network generators, and simulation tools. The proposed X-AS internet topology map has the advantage of comparing with three research networks and nine large-scale commercial network topology maps to determine the level of discrepancy between the X-AS map links and AS level map relations of the aforementioned sources. The experimental results showed that the X-AS map accurately captures the cross-border interface node fittings to research and commercial networks and covers the links between ASs. Furthermore, Nur et al.^19^ proposed an AS traceback packet marking algorithm based on the IP protocol record route option for inferring AS level forward route from malicious users toward a victim site. To investigate the average number of packets, the proposed algorithm was compared to two previous works conducted by Nur et al. and Yaar et al. Results indicate that the proposed AS traceback scheme employs 41–276 bits on average for fluctuating AS level hop distance. In terms of hop counts, the total average is 96.91 bits. Security is critical for end-to-end communication scenarios in any network infrastructure. Policy-based security architecture was suggested in Ref.^20^ for securing communication in one AS software-defined networking (SDN) domain environment and then extended the architecture for multiple AS SDN domain environments. Furthermore, when the performance of the network switches is examined, throughput flow varies between four switches during the experiment. Furthermore, CPU usage is greater than the requirement for the proposed policy-based security application. Funel used multiple data sources to analyze Internet’s graph structure at the AS level. The author discovered that the overall trend of the average connectivity has increased for more than 10 years, and the size of the internet has roughly doubled^21^. An online platform called ASmoniTor was suggested in Ref.^22^ to preserve multiple network users’ privacy. The suggested ASmoniTor can work without complicated source code compilations compared to the Tor network or the Onion router. Furthermore, it can help network users reduce errors in the AS path selection by informing them of the users’ input in terms of the presumed setup of ASs. Finally, users can maintain their privacy because ASmoniTor provides them complete control over the information that network users want to input. Oh et al.^23^ studied topological properties that define the Korea Internet AS level topology. They drew data for Korea Internet AS level characteristics from three sources. The Korea Internet AS graph is highly disassortative and has strong connectivity among rich club nodes when compared to several power-law techniques. The proposed study had the advantage of comparing several techniques in terms of the node degree distribution with the proposed technique to prove the accuracy of the Korea Internet AS graph. In the last 10 years, Witono et al. conducted topological Internet studies on Indonesia’s internet AS level. They obtained information from two well-known global Internet data analysis sources: APNIC and CAIDA. According to the findings, the Indonesian Internet has grown linearly in terms of AS nodes and AS links over the last 10 years with 114 new AS numbers appearing each year^24^. Hoeschele et al. studied 5G properties in the internet core, in particular, the importance of internet exchange points (IXPs) for 5G use cases. They mentioned four use cases, their data requirement, and the impact of the use cases at the internet core^25^. Karlin et al. suggested a novel network anomaly identification approach called pretty good border gateway protocol (PGBGP) to provide distributed anomaly detection, such as a comparable security system for cryptographic approaches, which has a more reasonable affectation path. The following are the outcomes and effectiveness of the proposed approach. First, if located on the major 0.5% of ASs, PGBGP could reduce the impacts (containing 0.07–2% of all ASs depending on the attack type) of original AS and invalid path attacks. Second, they demonstrated that PGBGP is nearly as effective as a better security solution at preventing network vulnerabilities. Third, the PGBGP protocol does not necessitate a global change alteration to the traditional BGP protocol^26^. Matcharashvili et al.^27^ proposed BGP dynamics and stability for discovering observed BGP update time series for regularities to analyze BGP dynamics and robustness. They used a multivariate analysis in Ref.^27^ based on the Lempel and Ziv complexity measures as well as Mahalanobis distance to examine BGP update time series for more than four ASs. The analysis results show that for discrete durations where update dynamics were more consistent, they were more consistent as compared to the periods where they were more random. Furthermore, they discovered that each of the four BGP update time series shows an increase in the longitude of order for specific days, e.g., starting from September 8 to September 27 until October 9, 2011, which is similar to routing dynamics with a global impact.

## Proposed methods

### Autonomous system edge vulnerability estimation

In this section, we have provided a detailed explanation of the proposed method. The proposed method for the AS PE router vulnerability estimation is as follows.

#### Data collection

**Figure 2:**
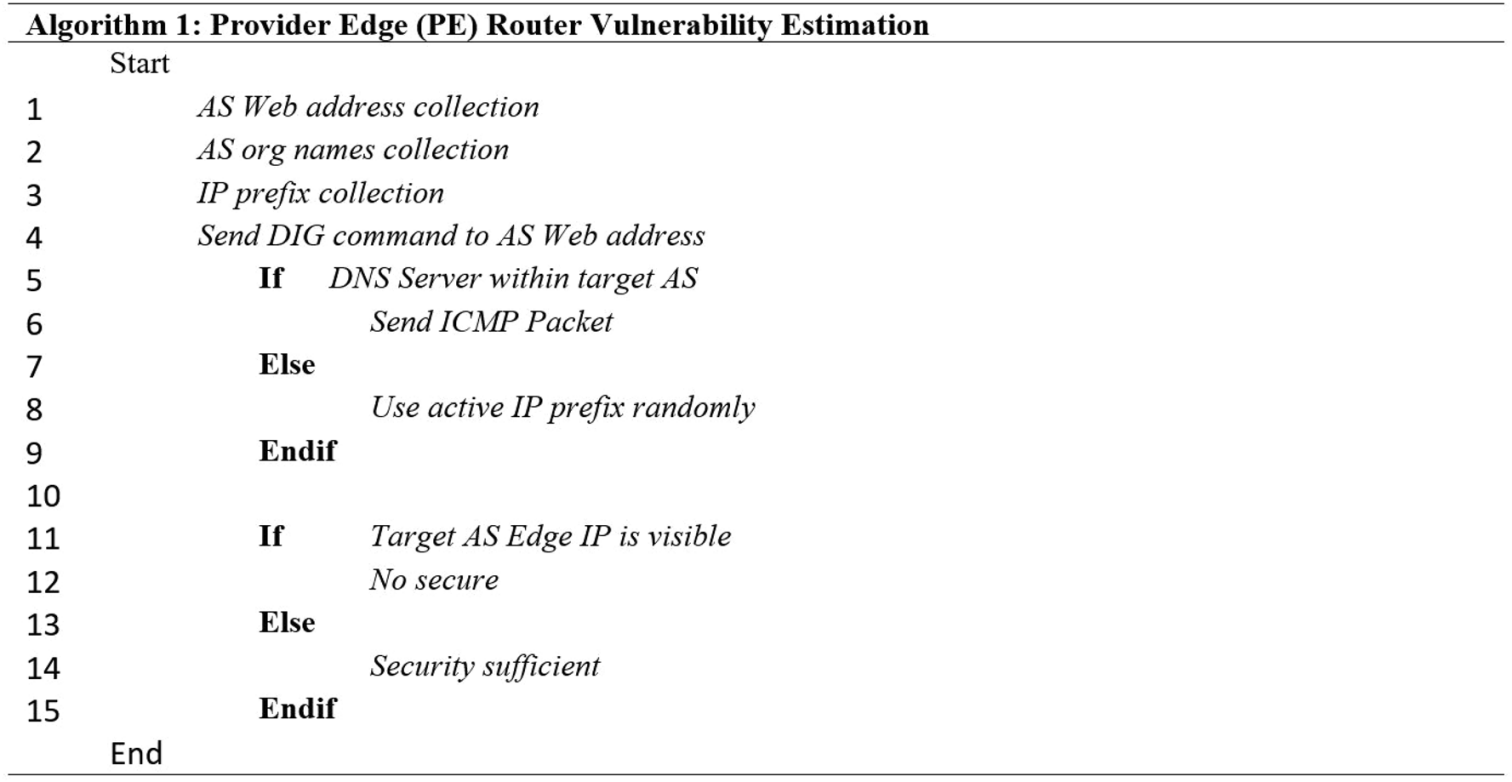
Proposed method for PE router vulnerability estimation.

First, we collected the required data from five data sources: Google search engine, CAIDA, Hurricane Electric Internet Services (HE), The Korea Internet and Security Agency WHOIS DB, and RIPE network coordination center (RIPE NCC)^28–31^. However, in this subsection, we have explained only the first four data sources which were mentioned in the first proposed method. Then, we have articulated RIPE NCC in the next subsection which is related to the second proposed method. Figure 2 shows the first proposed method where provide edge (PE) router vulnerability estimation is elaborated by means of an algorithm. Here first AS web address collection and AS organization name collection is done along with IP prefixing. After which a simple IF then Else based decision is made to check if IP is visible or not (which signifies whether a system is safe or not). From the Google search engine, the web addresses of the target AS related data were collected. Then, we gradually collected the data regarding target AS number and organizational names from CAIDA. Finally, we collected the target AS IP prefixes from the HE.

#### Sending domain information groper command to target autonomous system web address

In computer networking, domain information groper (DIG) is a network administration command-line tool for discovering DNS IP addresses of the target AS or any Internet organization. In this study, we employ the DIG command to collect all DNS IP addresses of the target ASs and send them ICMP packets. Through sending ICMP packets to the DNS IP address of the target AS, we realized that the PE router IP address of the target AS was visible or blocked by firewall software. We have considered that PE router IP address is the first IP address of any target AS where ICMP packets cross several hops from the source, which is our Computer in Kyungpook National University CCMP Laboratory until reaching the destination (which is DNS servers of the target ASs). We sent ICMP packets through the traceroute diagnostic command in the Linux OS terminal as *traceroute—ICMP target AS DNS IP*.

#### Checking geographical location of domain name system server via Whois DB

After finding the DNS IP address of the target AS through the DIG command-line in the LINUX OS terminal, the essential step is to identify the location of the DNS server. Checking the location of DNS servers is mandatory because of the installations of DNS services of some ASs were found from content delivery networks (CDNs). However, CDNs can provide fast and secure networking services due to their intelligent edge platforms and advanced networking infrastructure. Similarly, making a separate data center in every country is impossible due to the economic and social conditions of undeveloped countries. Therefore, we used the Korean Internet and Security Agency WHOIS DB to check whether the DNS server geographically belongs to the target AS or not. If DNS server is situated in our target AS that belongs to the specific country or continent, we have sent an ICMP packet to check the vulnerability condition of the target AS PE router. However, if we found the location of the target AS DNS server from vise verse, we sent an ICMP to a specific IP prefix of the target AS which were collected from HE DB.

#### Decision making regarding provider edge router vulnerability condition

**Figure 3:**
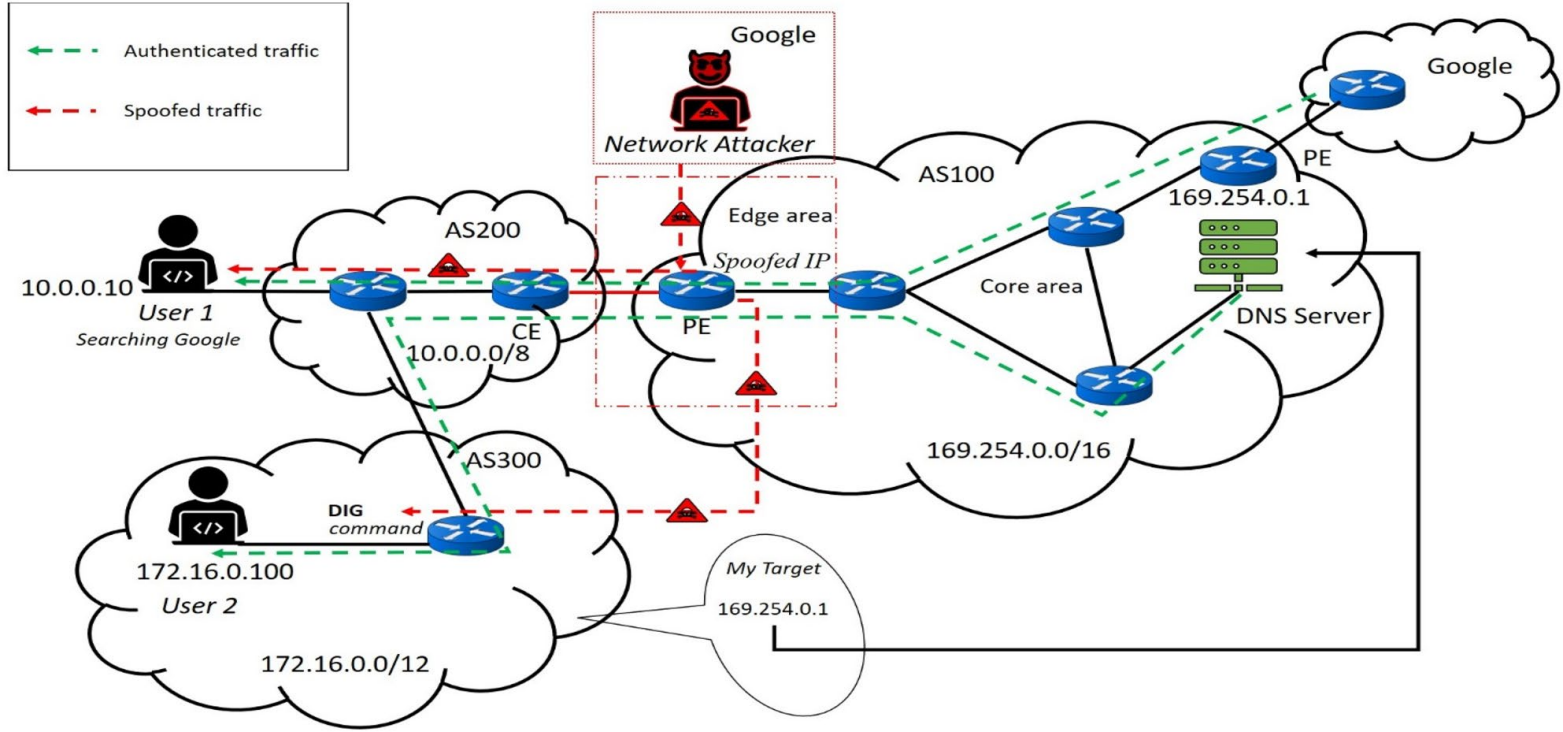
Example of PE router security essentiality.

Finally, sending ICMP packet either to the DNS IP address or any IP prefix of the target AS, which is active, is an appropriate method for decision making regarding the target AS PE router vulnerability condition. Understanding PE router vulnerability can be decided when the first IP address of the target AS is exposed while tracing several hops from the source to the destination. Unlike, security sufficient of the target AS PE router can be estimated, when ICMP traverse the first hop, the first IP address of the target AS if block and display the star {*} by the firewall program. We have decided that the PE router of the target AS is not vulnerable and protected by a firewall, which is essential for scanning outcoming nonauthorized traffic on the Internet. Additionally, PE router security is essential within a physical network infrastructure. PE router is located in the network boundary connected to the CE router in the transit ISP to provide data access for its neighboring ISP or customer ISP. Currently, there is a possibility of DDOS attacks within the network due to the increasing high-level network experts and illegitimate users. For instance, as shown in Fig. 3, the network attacker from the other unknown AS or outside network impersonates the legitimate user in AS200. In this scenario, the network attacker pretends to be a Google search engine and targets the PE router in AS100 to create malicious traffic for the legitimate user in AS200. Similarly, another home network user from AS300 plans to discover the DNS IP address of AS100, which is 169.254.0.1. However, a network attacker from another network spoofs the AS200 PE router IP address. In that case, the PE router IP address will be incorrect, and the reason for not authorized access to another network without the permission of a specific network administrator is a crime. As a result, one of the primary goals of this research is to expose the number of vulnerable PE routers in 600 AS around the world. Another critical issue that must be addressed is that we do not know the vulnerability state of all AS PE routers in the world based on the ICMP packet inspection method. As a result, we randomly selected several ASs from six continents to estimate their PE vulnerability by sending ICMP packets. The ASs which we have studied in this research are well known in terms of global data sharing for small-sized ASs and play an important role in regional data access.

### Autonomous system neighbor value prediction

**Figure 4:**
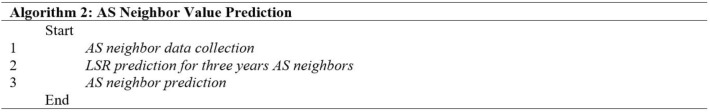
AS neighbor values prediction based on ML approach.

AS neighbor values are critical for implementing a decentralized network on the Internet. To the best of our knowledge, a decentralized network ecosystem can be analogous to the ASs with more neighbors and complex topologies. Notably, ASs in the form of centralized networks is more vulnerable to DDoS attacks than ASs in the form of compared decentralized networks. Furthermore, in the case of finding the AS neighbors that are decreasing rapidly as time passes, we considered such ASs as having a vulnerability risk. As a result, using the LSR statistical approach, we predicted the neighbor values of ASs for the next 3 years. The overall process of the proposed method for AS neighbor value prediction technique is illustrated in Fig. 4.

#### Data collection

The authors of this study used one of the well-known routing information registry (RIR) platforms called RIPE Network Coordination Centre (RIPE NCC) to collect target AS neighbor data^31^. The RIPE NCC can provide a variety of data sources for the analysis and measurement of global internet ASs. AS neighbor-related data from the RIPE NCC website was collected through analysis, internet measurement, and finally selecting routing information service (RIS). When the RIS section is selected, the ASN neighbors widget will display information about neighboring ASNs. The authors of this research collected 3 years of AS neighbor value data starting from January 2018 until August 2021. The actual goal of the data collection is to forecast the number of AS neighbors’ dynamism over the next 3 years, from 2022 to 2024, using a machine learning (ML) approach.

#### Forecasting based on a machine learning approach

Following the completion of data extraction from RIPE NCC/RIS, the author used the LSR statistical approach to predict the desired outcome using a Microsoft Excel spreadsheet formula called *Forecast*. LSR is a statistical computation technique that can forecast a response variable outcome using multiple explanatory variables. Notably, one of the benefits of using Microsoft Excel is convenience and time saving. It is effective for computing large and complex datasets based on ML algorithms. Using the multiple prediction trendline option of explanatory variables improved the clarity and understanding of our prediction.

## Experiments and results

This section highlights the study and experimental setup’s contributions.

### Implementation details

During the experimental setup, we used a large number of computer components, operating systems, and tools to achieve accurate results for estimating vulnerability in 600 AS PE routers. For tracerouting to various locations around the globe, we have used a network tool from the Linux OS that was installed on VMware 16 virtual machine. VMware was run in a 64-bit Windows 10 Educational Operation System by utilizing an Intel (R) Core i5-6500 (6th Generation) CPU with the speed of 3.20 GHz, 16 GB RAM, B150M Pro5 mainboard, and NVIDIA GeForce GTX 1050 Ti Graphic card to perform several tasks. Moreover, the authors of this research used a variety of Python libraries to perform AS characteristics analysis such as Matplotlib, NumPy, and Pandas. Using MS Excel program was more convenient for predicting AS neighbor values. As a result, we utilized the MS Excel prediction formula to perform the required prediction of AS neighbor values. Table 1 provides a detailed overview of the implementation details.

**Table 1:** Implementation properties.

No.	Component names	Details of the component
1	Motherboard	B150M Pro4
2	CPU	I5 6500 Gen and 3.20 GHz frequency
3	RAM	16 GB and DDR4 1066 MHz frequency
4	SSD	500 GB capacity
5	GPU	4 GB capacity

No.	OS and tools	Details of the OS and tools
1	Windows 10 64 bit	16 GB RAM
2	Ubuntu 64 bit 20.04 for Linux	8 GB RAM
3	Python Programming Language	Matplotlib, NumPy and Pyplot libraries
4	MS Excel	Forecasting formula

#### Estimation of PE routers vulnerability and security sufficient condition

**Figure 5:**
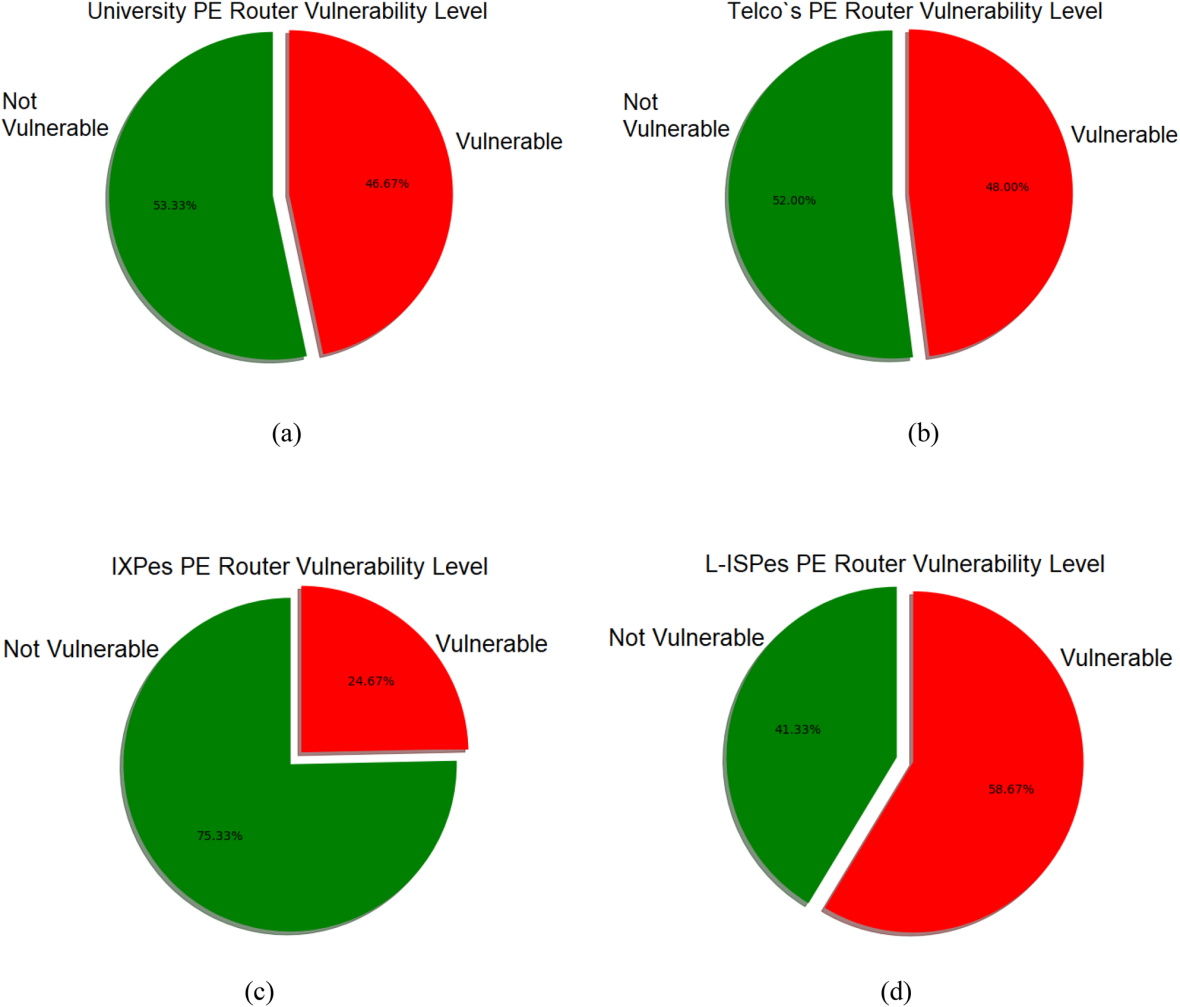
Different types of AS PE router vulnerability estimation.

For checking the security condition of 600 global ASs, we have demonstrated them into four categories. Each AS type is shown in the pie graphs to represent the percentage number of visible IP addresses that are vulnerable and not visible IP addresses, which are secure. We have demonstrated them into 150 global Universities, 150 Mobile Companies, 150 IXPs, and 150 Large ISPes. As shown in Fig. 5a, the number of vulnerable PE router IP addresses contains 70 into red colors, equal to 46.67% of the 150 University ASs. The remaining 80 IP addresses were found to be secure in green color (53.33%). Similarly, in Fig. 5b, the number of vulnerable PE router IP addresses contains 72 into red colors, equaling to 48.00% of the 150 Mobile Company ASs. The remaining 78 IP addresses were found as secure in green color (52.00%). As a result, we found almost the same category of vulnerable IP addresses within Universities and Mobile Companies. Correspondingly, while checking IXP PE router security condition in Fig. 5c, the number of vulnerable PE router IP addresses contain 36 indicates in red color, equal to 24.00% of the 150 IXP ASs. The remaining 114 IP addresses were found as secure in green color (76.00%). The reason for less vulnerable PE router IP addresses is that IXPs are important for other global ISPs for providing direct interconnection without third party ASs. The key benefits of direct interconnection are reducing the price of traffic, network latency, and bandwidth. Therefore, the quality of IXP network switches comparatively should be better than other physical network infrastructure from the network product viewpoint. Finally, in Fig. 5d, the number of vulnerable PE router IP addresses is 88 indicated by red color, equal to 58.67% of the 150 Large ISP ASs. The remaining 62 IP addresses were found to be secure indicated by green color (41.33%). The main mission for analyzing the specific AS PE router vulnerability condition is that the collected 600 ASs are globally important and well known. Additionally, minority of these ASs is located in the Internet core. Therefore, network security engineers must protect the edge network of the above global AS networks. The reason for using ICMP packet in this study is that, by comparing to other network diagnosis methods, it is convenient to estimate the AS PE router vulnerability. However, the number of DDOS attacks is increasing globally. ICMP not only stands for diagnosing the network condition but also DDOS attacks.

**Figure 6:**
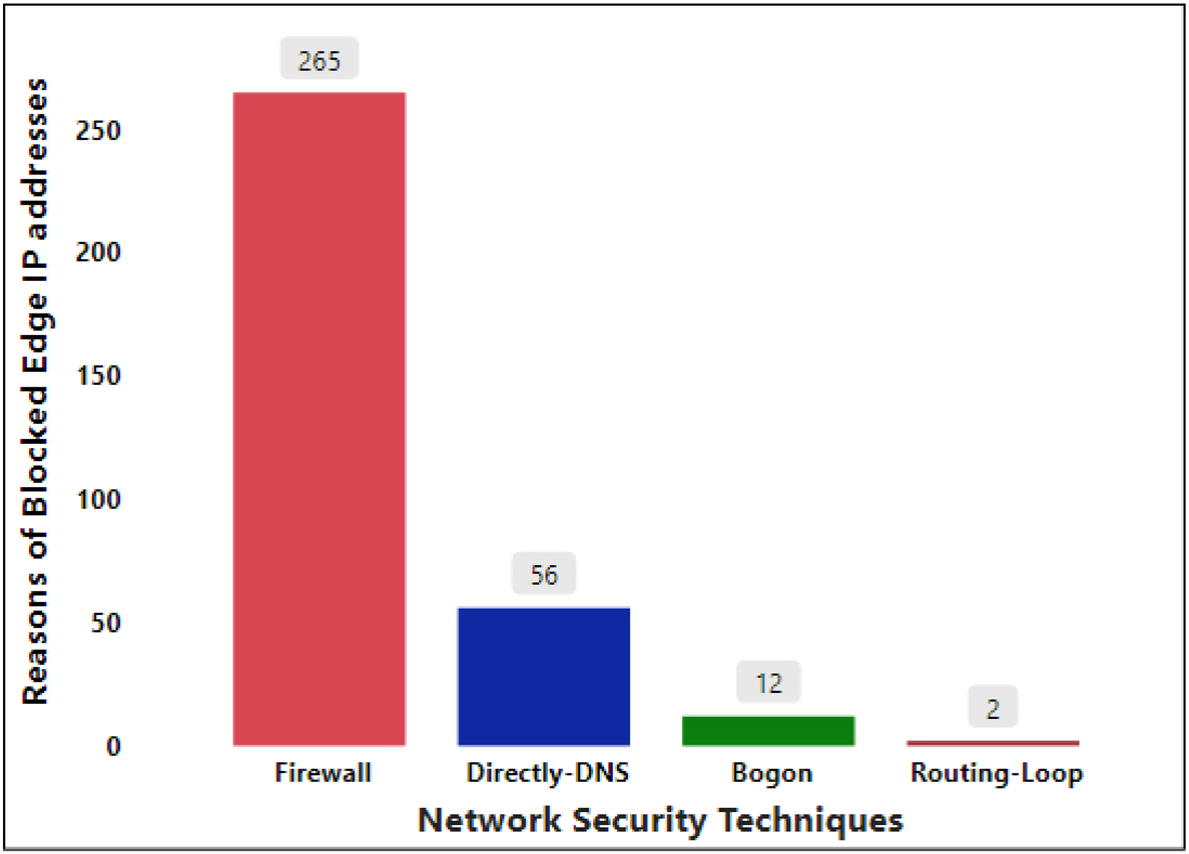
Classification of ASs that are not vulnerable through ICMP.

To estimate the number of PE router security techniques employed by ASs and not vulnerable due to ICMP, the total number of such ASs is shown in Fig. 6. The total number of ASs, which PE router IP addresses were not vulnerable through ICMP, is equal to 335 from the total of 600.

Among 335 ASs that do not have visible IP addresses through ICMP, 265 ASs were blocked by a firewall program; 56 of the transmitted packets were reach directly to the DNS IP address, which is the target of every AS. Due to the direct jump of ICMP packets to the DNS server, it was impossible to discover edge IP addresses. Bogon or Bogon filtering are fake IP addresses of a computer network that are not held in any specific range allocation by the Internet Assigned Numbers Authority (IANA)^32^. 12 ASs were not vulnerable through ICMP due to Bogon sharing on the public Internet. Finally, two AS edge IP addresses were not available through ICMP due to a routing loop issue. The computer networking routing loop is a well-known issue that occurs due to the confusing shortest path to the destination by several nodes^33,34^. Accordingly, during global AS characteristic analysis, we discovered that some PE routers were secured through network security techniques and some routers had the problem of misconfiguration.

### Overview of the AS neighbor value prediction process

To predict the number of 600 AS neighbors, we selected LSR as a convenient approach. As shown in the bottom general parametric equation (Eq. (2)), it is possible to predict multiple explanatory variables; using the linear forecasting method in MS Excel, we could identify the number of future AS neighbors.

2 $${Y=\beta }_{0}+{\beta }_{1} {\user2{X}}.$$

Here, ${\varvec{a}}$ is an intercept of LSR and the point where the trendline crosses toward the steepness or slope of the function. As the steepness increases, there is a high probability of AS neighbors increasing. *X* are explanatory variables (independent), which we try to predict. β is the slope of the function, which is the path and steepness of the trendline. *E* is the error term describing the expected outcome at the specific time and the observed result. Equation (3) indicates the mean of LSR computations form, which can be expressed as follows:

3 $${\varvec{\mu}}=\frac{\boldsymbol{\Sigma }{\varvec{X}}}{\boldsymbol{\rm N}}.$$

We can perceive $\boldsymbol{\Sigma }$ as the sum value of explanatory variables, where *X* is expressed as an explanatory variable. *N* is the total number of explanatory variables in row form which can be divided into the sum of explanatory variables. Finally, ${\varvec{\mu}}$ express the output of the mean of the LSR function; it is illustrated in Eq. (4) and used for finding the slope of the prediction line first. After defining the mean, we subtracted all explanatory variables from their mean in the form of $\left({\varvec{x}}\right.-\overline{{\varvec{x}} }$). The same process was applied for ${\varvec{y}}$. After this process, we have to find their square one by one as $\left({\varvec{x}}\right.-\overline{{\varvec{x}} }$)^2^.

4 $${{\varvec{\beta}}}_{1}=\frac{\boldsymbol{\Sigma }\left({\varvec{x}}\right.-\overline{{\varvec{x}} })\times ({\varvec{y}}-\overline{{\varvec{y}} })}{\boldsymbol{\Sigma }\left({\varvec{x}}-\overline{{\varvec{x}} }\right){\textbf{2}}}.$$

First, finding ${{\varvec{\beta}}}_{1}$ value (slope) is important to compute ${{\varvec{\beta}}}_{0}$. Foremost, the slope value ${{\varvec{\beta}}}_{1}$ is multiplied by the sum of X vales then subtract by Y values. Lastly, the value which was derived it must be divide by the *n* values of X and Y. The Eq. (5) is indicated Y-intercept or ${{\varvec{\beta}}}_{0}$.

5 $$\frac{{{\varvec{\beta}}}_{0}=\boldsymbol{\Sigma }{\varvec{Y}}-{{\varvec{\beta}}}_{1\boldsymbol{ }}\boldsymbol{\Sigma }{\varvec{X}}}{{\varvec{n}}}.$$

It is worth noting that in some studies, LSR parameters are different. However, in this study, we have mentioned a general name of all LSR parameters which is used in statistics.

#### Experimental process for AS neighbor values prediction

The order of global ASs characteristic analysis started from Universities, Mobile Companies, IXPs, and Large ISPs from the Introduction and other sections. Therefore, we first explain the future neighbor values of the University type ASs. The total number of Universities was 150 and we have demonstrated them using four graphs. The first three graphs contain 37 AS neighbor value predictions. However, the last graph contains 39 AS neighbor values. The total number of entire ASs of every category is 150. Such distribution is shown through Excel programming plots; it was not possible to visualize 150 prediction trendlines of LSR. Even by showing separate plots containing 37 ASs, most AS neighbor value prediction trendlines are not visible, because a significant number of ASs are similar in terms of neighbor value in the last 3 years. Figure 5a only indicates 11 trendlines, because the remaining trendlines are superimposed onto each other. However, in Fig. 5b–d, majority of forecasting trendlines are superimposed on each other and hidden. As shown in the Y-axis, every AS neighbor values are significantly different due to the dissimilar neighbor values among ASs. For instance, many negative relationship cases of AS neighbor values were found in the future AS neighbor values that are dramatically decreasing or will disappear as Internet companies from the global web.

**Figure 7:**
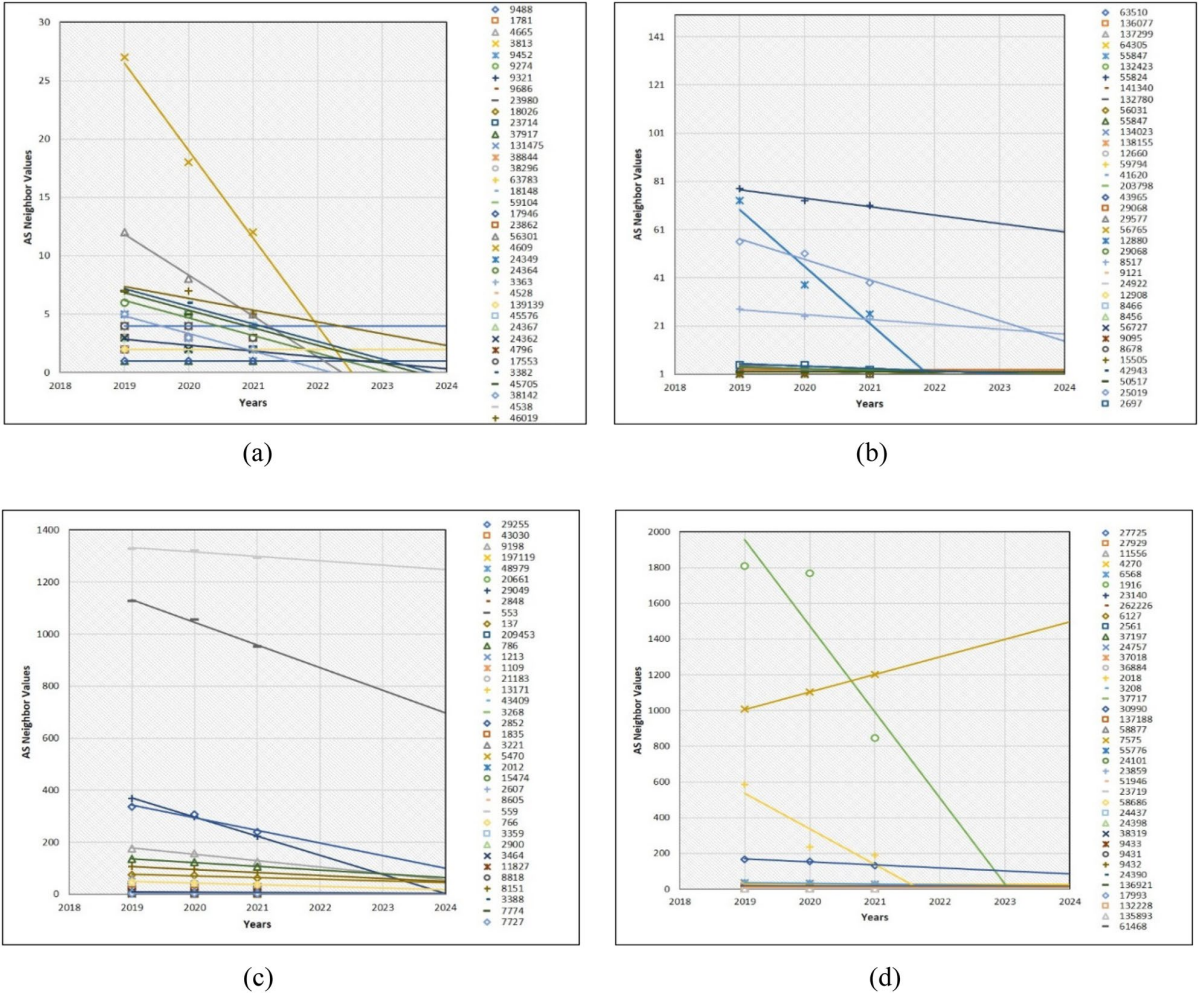
Prediction of 150 university type AS neighbor values.

Additionally, there are many common cases in which AS neighbor values will dramatically decrease in the next 3 years. It is possible to see such cases in Fig. 7a AS3813, Fig. 7b AS12880, Fig. 7c AS29049, and Fig. 7d AS1916 and AS2018. We have provided all AS numbers on the positive side of every figure. Thanks to MS Excel, it is feasible to differentiate the entire AS number values with their unique symbols. Table 2 presents the reason for decreasing AS neighbor values as follow.

**Table 2:** Reasons of AS neighbor dynamics.

No.	Reasons of AS neighbor values
1	Due to price of traffic to find low-cost ISP
2	Due to change of routing path for finding adjacency path
3	To have two or more upstream ISP so by the passage of time due to bill of traffic one or any of them can be cut off
4	Due to disappear of some company, which initially used Internet access as a network access necessity for his/her company

##### Due to the price of traffic to find low-cost ISP

The cost of ISP traffic is a critical consideration for its customer ISPs. Customer ISPs, on the contrary, usually try to find their providers not only because of the low traffic for the monthly price but also because of the Internet speed range.

##### Due to the change of routing path for finding adjacency path

**Figure 8:**
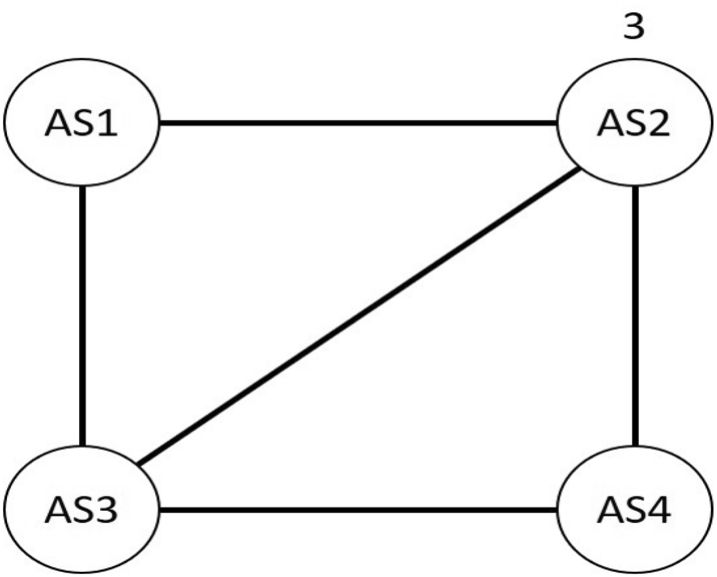
An example of four ASs with their adjacency path.

Another reason for decreasing AS neighbor value is finding adjacency path by the passage of time. The example of finding an adjacency path for AS neighbors are similar to transforming the adjacency matrix into graph form as illustrated in Fig. 8. AS2, for example, has three adjacent neighbors due to its connected edges, whereas AS1 has only two adjacent neighbors and cannot connect directly to AS4 due to adjacency issues.

In the adjacency matrix, we must consider the matrix rows and columns. We labeled the AS1–AS4 as Labels 1, 2, 3 and 4 in the adjacency matrix form. We refer to the adjacency matrix as A. The adjacency matrix’s formal definition is as follows:

6 $$ A_{k,l} = \left\{ {\begin{array}{*{20}ll} {1,} &\quad {if\; k,l \; \in \; E} \\ {0,} &\quad {otherwise} \\ \end{array} } \right.. $$

As it is expressed, if *k* and *l* are connected, it is an actual edge. However, if *k* and *l* are distant, they are not an edge. The primary significance of defining adjacency in the matrix form is that if the values are adjacent or connected, it is indicated as 1. However, if the distant numbers are expressed as 0 or loop, they are indicated by the bottom matrix.7

Any value in the adjacency matrix form is connected and considered as an adjacent vertex; the same thing is applied in the Internet ASs, which is why finding adjacent of decreasing neighbors reduces their distant neighbors. Thus, the adjacent AS neighbor is efficient for solving load balancing on the Internet.

##### Possessing two or more upstream ISPs by the passage of time due to bill of traffic some of them can be cut off

Some ASs typically have two or more ISPs to provide Internet access to their customers. However, as time passes and the cost of Internet traffic rises, any one of them will be forced to terminate the contract with the customer AS. Such an example can occur in the University-type ASs. For example, if the number of applicants is reduced in specific semesters, the University’s budget will be reduced; thus, there should be some solution to reduce the burden of decreasing budget.

##### Due to the disappearance of some companies, which initially used Internet access as a network access necessity for his/her company

**Figure 9:**
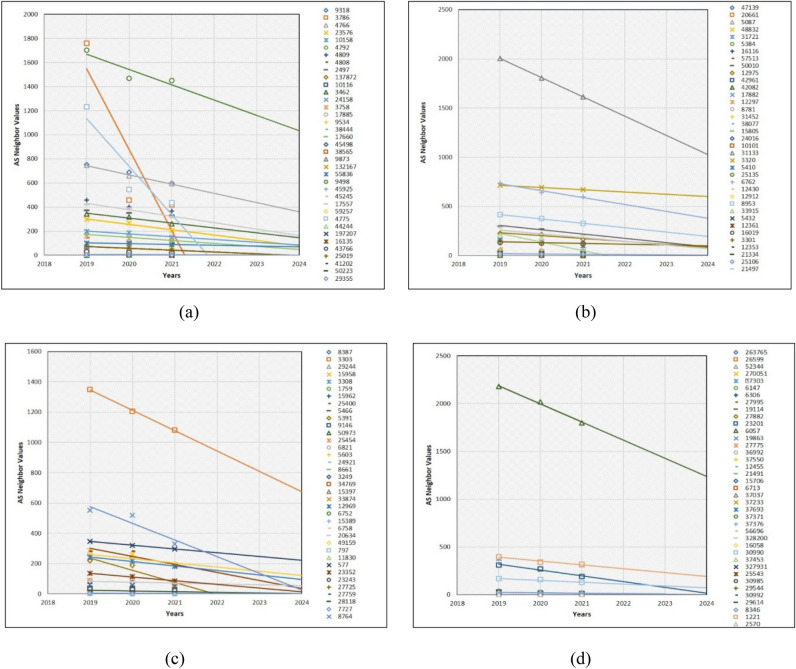
Prediction of 150 Telco type AS neighbor values.

Some small-sized business companies are buying traffic from particular ISPs to have reachability to the Internet access for their teams. In general, such businesses will continue to exist if their plans are successful; otherwise, they will disappear as small- and medium-sized enterprises and be the cause for decrease of some AS neighbor values. Similarly, the same case occurs during the prediction of the telecommunications company (Telco)-type AS neighbor values for the next 3 years (Fig. 9). Especially, AS3786 and AS4775 neighbor values are dramatically decreasing in Fig. 9a. However, AS31133, AS21497, AS6762 and AS33915 in Fig. 9b, AS8764, AS3303 and AS5391 in Fig. 9c, and AS1221 and AS6057 in Fig. 9d are examples of the Internet AS neighbor values that are significantly decreasing. Remaining AS neighbor values which are not visible as a trendline in the plots are superimposed. Usually, such AS neighbors have two to five neighbor values. Additionally, one of the drawbacks of our prediction is that we could not completely visualize the entire trendline in each graph. Another mandatory point for the 16 prediction graphs in the experiments and results section is that scatter dots in each graph are the AS neighbor values. Moreover, the trendline without the scatter dots is the prediction line. If the prediction line is steep, it means the AS neighbor values are increasing and depends upon the level of the line steepness. However, in the case of the decline, it expresses the reduction of AS neighbor values. Furthermore, we found that some AS neighbor values in the last 3 years was in constant condition, which is the outcome of the prediction which will be constant as well.

**Figure 10:**
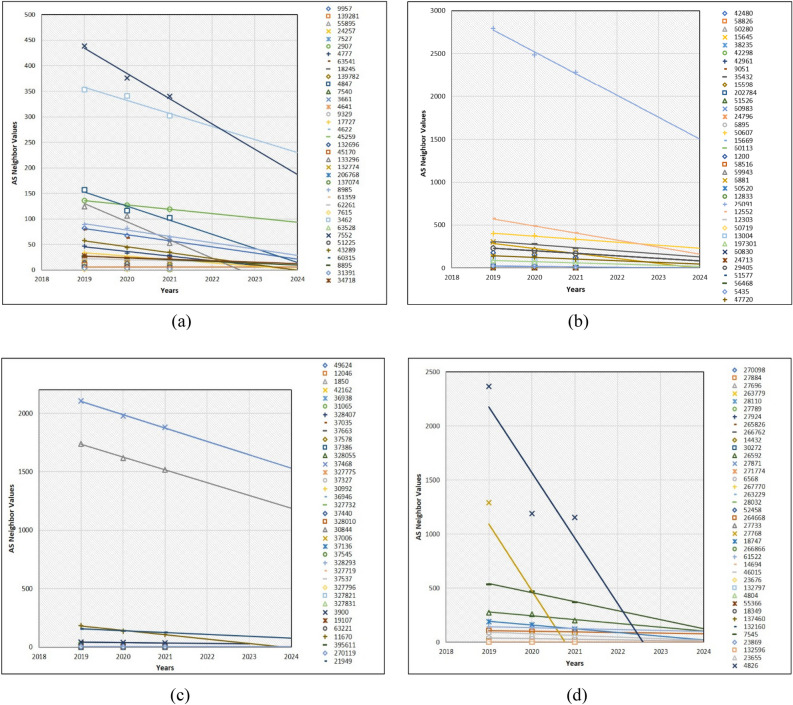
Prediction of 150 IXP-type AS neighbor values.

As shown in Fig. 9, in the future, some specific number of IXP type AS neighbor values on the level of the line are decreasing. Notably, AS7552, AS3462, AS4847, and AS133296 in Fig. 10a, AS25091, AS12552, and AS6881 in Fig. 10b, AS37468, AS1850, and AS11670 in Fig. 10c, and AS4826, AS27768, and AS7545 in Fig. 10d are significantly decreasing. The prediction of IXP type AS neighbor values is that the majority of AS neighbor values are superimposed as they have less value or the same values.

**Figure 11:**
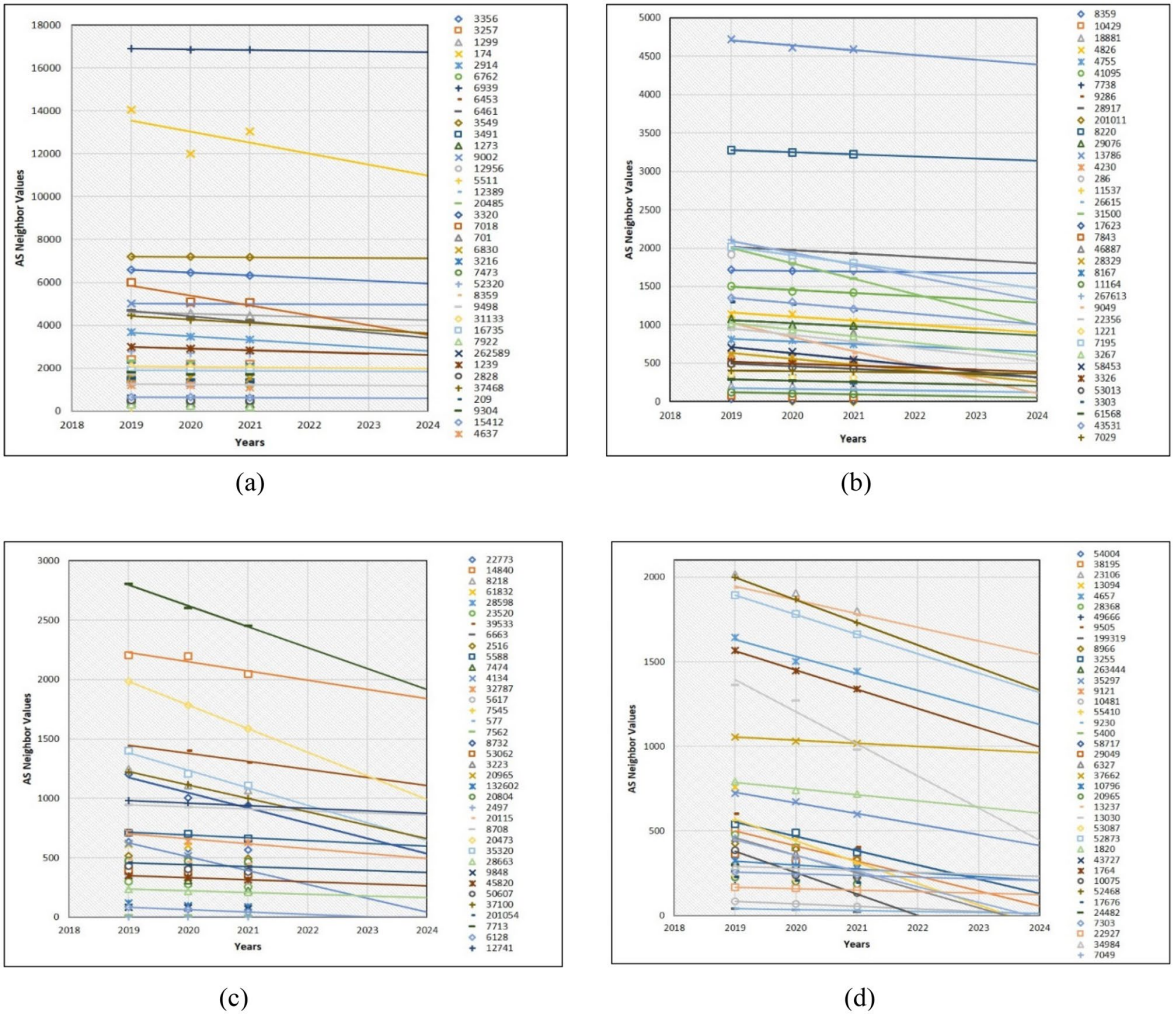
Prediction of 150 L-ISP-type AS neighbor values.

After predicting the AS neighbor values of large ISPs, we discovered that the number of AS neighbor values that are decreasing is less. However, such AS neighbor values, which were decreasing, were numerous in the above three AS categories. This is because 150 large ISPs investigated in this study are located in the global internet core. Therefore, they are essential for global Internet P2P and P2C services. Additionally, their neighboring values will either constantly stay to provide Internet access to the global Internet edge or other novel intermediate ISPes will be connected to them. AS174 and AS7018 in Fig. 11a indicate that their neighbor values are slightly in the declining condition. The remaining 35 AS neighbor values are nearly identical and are expected to remain constant, because we discovered that some AS neighbor values were in constant condition over the last 3 years. Unlike, in Fig. 11b, AS13786 neighbor values also will be slightly reduced. Similarly, AS31500, AS7195, AS267613, and AS9049 are significantly predicted to be reducing. The same condition, but dramatically, is predicted to happen on AS7713, AS53062, AS20473, AS39533, AS35320, AS37100, AS8732, AS32787, AS4134, and AS6128 in Fig. 11c. The remaining AS neighbor values fall under the category of very slightly decreasing or staying constant. The problem that occurs frequently is that while predicting some AS neighbor values, some prediction trendlines are superimposed due to having the same AS neighbor value. Finally, in Fig. 11d, 13 AS neighbor values including AS13237, AS52468, AS6367, AS52873, AS10796, AS1764, AS13030, AS1820, AS35297, AS3255, AS55410, AS38195, and AS10075 are forecasted to decrease dramatically. For example, AS neighbor values of large ISPs in all the four graph trendlines are more visible than other AS neighbor value prediction trendlines within graphs. This is because the large ISP neighbor values are numerous than other AS neighbor values. The overall AS type neighbor values that are not mentioned in this have the same neighbor value and are superimposed.

In summary, it is essential to state that data collected for large ISP neighbor values are according to the order of CAIDA AS rank, compared to the order of the above AS categories. This is because CAIDA’s AS rank indicates the AS according to AS degree or relationship. For instance, if AS1 has more degrees than AS2, the AS rank of AS1 is 1 and AS2 rank is 2. The large ISP type AS neighbor values are the Internet backbone and must be selected according to CAIDA AS rank rather than based on personal choices from different locations of the globe.

## Conclusion

The global Internet infrastructure is growing progressively. In this study, we proposed a method to insight and analyze the number of vulnerable AS PE routers through ICMP packets. We collected 600 AS names categorically dissimilar from the different locations of the globe. After experiments, we obtained that 265 of the 600 global ASs were under the ASs whose PE routers are vulnerable through ICMP. Edge or PE routers are important network components within Internet AS. It is not possible to use data on the Internet effectively without a secured PE router. If PE routers are not securely protected by the special firewall software or firewall hardware or any other data communication protection techniques, there will be malicious attacks by the network attackers from incoming network traffic. Based on this fact, network AS found vulnerable by ICMP must install firewall software or firewall hardware components in their networks to scan network traffic while utilizing network users. We provided information for the number of unsecure AS types through ICMP packets collected and categorized them into pie charts using ordinary statistical techniques. Additionally, we provided the next three-year prediction based on LSR for the entire AS neighbor values, change with time or sometimes stay constant. As a future study, we will investigate the new challenges and upcoming security issues of the future generation internet and security issues of CDN edges.
